# Noise during Rest Enables the Exploration of the Brain's Dynamic Repertoire

**DOI:** 10.1371/journal.pcbi.1000196

**Published:** 2008-10-10

**Authors:** Anandamohan Ghosh, Y. Rho, A. R. McIntosh, R. Kötter, V. K. Jirsa

**Affiliations:** 1 Theoretical Neuroscience Group, Institut des Sciences du Mouvement, Marseille, France; 2 UMR6233, CNRS, Marseille, France; 3 Center for Complex Systems and Brain Sciences, Physics Department, Florida Atlantic University, Boca Raton, Florida, United States of America; 4 Rotman Research Institute of Baycrest Center, Toronto, Ontario, Canada; 5 Department of Cognitive Neuroscience, University Medical Centre St. Radboud, Nijmegen, The Netherlands; 6 Vogt Brain Research Institute and Anatomy II, Heinrich Heine University, Düsseldorf, Germany; University College London, United Kingdom

## Abstract

Traditionally brain function is studied through measuring physiological responses in controlled sensory, motor, and cognitive paradigms. However, even at rest, in the absence of overt goal-directed behavior, collections of cortical regions consistently show temporally coherent activity. In humans, these resting state networks have been shown to greatly overlap with functional architectures present during consciously directed activity, which motivates the interpretation of rest activity as day dreaming, free association, stream of consciousness, and inner rehearsal. In monkeys, it has been shown though that similar coherent fluctuations are present during deep anesthesia when there is no consciousness. Here, we show that comparable resting state networks emerge from a stability analysis of the network dynamics using biologically realistic primate brain connectivity, although anatomical information alone does not identify the network. We specifically demonstrate that noise and time delays via propagation along connecting fibres are essential for the emergence of the coherent fluctuations of the default network. The spatiotemporal network dynamics evolves on multiple temporal scales and displays the intermittent neuroelectric oscillations in the fast frequency regimes, 1–100 Hz, commonly observed in electroencephalographic and magnetoencephalographic recordings, as well as the hemodynamic oscillations in the ultraslow regimes, <0.1 Hz, observed in functional magnetic resonance imaging. The combination of anatomical structure and time delays creates a space–time structure in which the neural noise enables the brain to explore various functional configurations representing its dynamic repertoire.

## Author Summary

There has been a great deal of interest generated by the observation of resting-state or “default-mode” networks in the human brain. These networks seem to be most engaged when persons are not involved in overt goal-directed behavior. These networks are also thought to underlie certain aspects of conscious introspection and to be specific to humans. Our paper provides a new explanation for rest state fluctuations by suggesting that they reflect a deeper biological principle of organization and are a consequence of the space–time structure of primate anatomical connectivity. In a computational study using a biologically realistic primate cortical connectivity matrix, we show that the rest state networks emerge only if the time delays of signal transmission between brain areas are considered. The combination of anatomical structure and time delays creates a space–time structure in which the neural noise enables the brain to explore various functional configurations representing its dynamic repertoire. The latter repertoire spans temporal scales of multiple orders of magnitude including scales observed in electric potentials and magnetic fields on the scalp, as well as in blood flow signals. Our results provide a testable explanation of the real-world phenomenon of rest state fluctuations in the primate brain.

## Introduction

When subjects are not actively engaged in goal-directed mental activity, spontaneous brain activity has been suggested not to simply represent “noise”, but rather implicate spontaneous and transient processes involved in task-unrelated imagery and thought [1]–[9]. The resting state networks that are not associated with sensory or motor regions have been thought of as a “default-mode” network specific for the human and include medial prefrontal, parietal, and posterior and anterior cingulate cortices [4],[5]. Recent results by Raichle and co-workers [10] showed similar networks in monkeys during deep anesthesia suggesting that this default-mode network is, first, not specific for the human and, second, that it transcends levels of consciousness. Furthermore, the assumption of a link between resting state activity and mental processes is founded largely “ex negativo” upon Positron Emission Tomography (PET) and fMRI studies showing the deactivation of the “default-mode” network in correlation with the increase in task-related activity in sensory-driven areas during goal-directed behavior. The dynamics of these spontaneous fluctuations evolves on a slow time scale of multiple seconds. On faster time scales of 10–500 ms, EEG and MEG identify characteristic oscillatory modes of brain activity showing transient spindle like behaviors, which repeat themselves intermittently. These wave patterns are strongly dominated by alpha waves (8–12 Hz) when subjects have their eyes closed, and weaker but still clearly present for eyes open condition. In contrast to the well-studied phenomenology of alpha waves, no firm explanation exists regarding their genesis [11]. Similarly, since the first report of coherent rest state fluctuations observed in fMRI by Biswal et al. [1] more than 10 years ago, the mechanism for their generation remains poorly understood. Most hypotheses on the underlying mechanisms of rest state dynamics in the EEG/MEG consider alpha wave generation and postulate either of two hypotheses: pacemaker oscillators in the thalamus or cortex generate rhythms endogenously, which entrain the remainder of the cortex [12],[13]. Alternatively, the neural resting state activity arises from the network interactions of the cortex and thalamus. For the latter hypothesis, the neuronal network may either act as a narrow band transmission system (i.e., as a filter originally proposed by Prast in 1949 [14]) receiving white noise as input and producing the irregular rhythms; or the neural network generates a purely deterministic, often chaotic, signal reflecting the dynamics of coupled nonlinear oscillators [15]–[19]. All these computational models have some experimental support, but in general are too vague to pinpoint specific mechanisms. None of these models so far attempts to address the generation of the ultraslow oscillations observed in the fMRI. In a recent study by Honey et al. [20], chaotic oscillators representing neural population activity were linked mimicking the connectivity of the macaque. When computing the corresponding hemodynamic response signal from their simulations, the authors were able to reconstruct inter-area correlations found experimentally in the fMRI. Though the temporal dynamics of the hemodynamic response appears realistic on the ultraslow scale, their generating neural network model does not attempt to model faster electrophysiological rhythms as observed in EEG and MEG recordings. From these attempts it is evident, that there is currently no satisfactory explanation of the various phenomena related to rest state activity on multiple scales.

To shed light on the emergence of the resting state networks and their dynamics on various temporal scales, we performed a network simulation study in which the major ingredients were biologically realistic primate connectivity of brain areas, time delays via signal propagation between areas and noise. Our rationale is as follows: populations of neurons are dynamic systems capable of displaying oscillatory behavior. Imagine an isolated neural population that has no connections and is quiescent in the absence of noise. When noise is present, a fluctuation can perturb the population from its equilibrium state, to which it then returns in a characteristic transient manner. The latter transient will crucially depend on the “dynamic repertoire” of the population, which is the set of dynamic behaviors that a neural population can perform in the proximity of its equilibrium state. For instance, a damped mechanical pendulum is only capable of showing an oscillation with fixed frequency and exponentially decreasing amplitude following a perturbation, which defines its dynamic repertoire. Clearly, when neural populations are connected in a network, then the network connectivity will shape the dynamic repertoire of the entire network. Since the rest state networks are large scale networks distributed on spatial scales ranging up to almost 20 cm, the time delays via signal transmission between populations need to be considered. This can be understood intuitively along the following example: two systems shall oscillate in a synchronous fashion at 10 Hz. Their coupling shall be such that it reinforces synchronous in-phase oscillation when no signal transmission delay exists. If the delay increases to 50 ms, then the previously stable synchronous oscillation may become unstable, because the transmitted signal from one oscillator arrives now during the antiphase of the other oscillator. This example illustrates that the space-time structure of the couplings defined by the anatomical connectivity (space) and the time delays (time) will be the primary component shaping the dynamic repertoire of any large scale network. In the following we study these components systematically and evaluate their potential contributions to the emergence of rest state networks.

## Results

**Figure 1:**
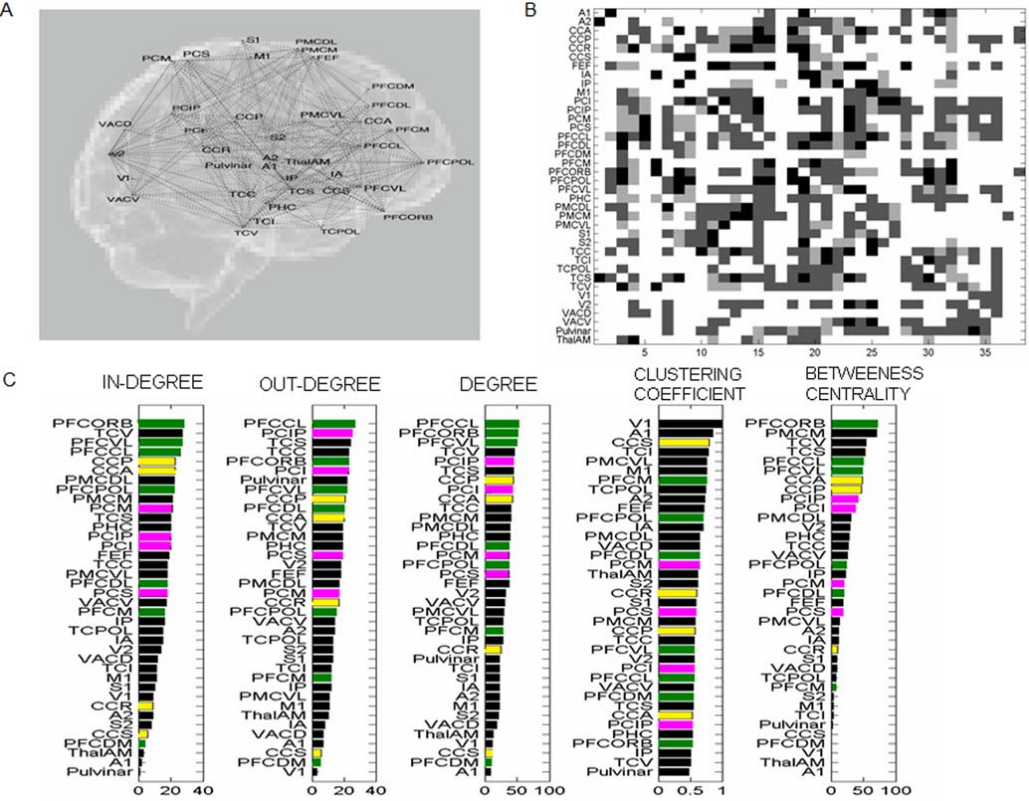
Anatomical connectivity. (A) The primate cortical connectivity map is overlaid over the human cortical locations. See Table 1 for abbreviations. (B) The connectivity matrix of primate cortical graph (white pixel implies no connection; grey scale indicates the weight of the connection). The columns are sources and rows are targets. (C) Statistical characterization of the primate connectivity matrix: node wise degree distributions, clustering index and betweeness centrality (14). The color codes for areas of interest: green, prefrontal; magenta, parietal; yellow, cingulate; black, all others. The connectivity matrix has characteristic path length = 1.633; clustering index = 0.568.

We first performed a graph theoretical analysis of the anatomical connectivity matrix of a single hemisphere obtained from the CoCoMac database [21]. Thus, we initially consider only the spatial aspect of the couplings. The connectivity matrix collated from macaque tracing studies comprises 38 nodes with weights ranging from 0 to 3 (see Figure 1; see also Table 1 for abbreviations). The corresponding “Regional map” gives the translation between macaque and human neuroanatomy [22]. It is to be noted that some connections between some areas are not known. For the subsequent simulations we assign random weights to these unknowns within the range of 0 and 1, but omit these in the graph theoretical analyses. The connectivity matrix is shown in Figure 1B, where the columns are targets and the rows are sources. To explore the connectivity characteristics quantitatively, we compute a set of network connectivity measures [20],[23] for all nodes including the in- and out-degree of connectivity, the clustering coefficient and betweeness centrality (see Methods) are computed on the binarized graph, and are shown in Figure 1C. When computing these measures for the weighted graphs, no significant differences are found. If the putative components of the resting state networks (see Table 2) differentiated themselves from other network nodes on the pure basis of anatomical connectivity, we would anticipate finding a clustering of these components in some of the graph theoretical measures, likely at the largest values. For better visualization, we use a color coding for the various components in Figure 1C. Anatomically, the prefrontal cortex is characterized by a large degree of afferent and efferent connectivity, whereas the temporal and medial parietal areas display only an intermediate degree of connectedness. Clustering index and betweeness centrality are commonly used to identify hubs in a network, but do not clearly differentiate the default network either, though the betweeness centrality measure shows a cluster of six components comprising prefrontal, parietal and cingulate cortices (PFCCL, PFCVL, CCA, CCP, PCIP, PCI) for midrange values. Based on this graph theoretical analysis, we find that pure anatomical connectivity of the large scale network does not suffice to reliably identify the network constituents during rest.

**Table 1:** List of cortical areas.

Abbreviation	Cortical Area
‘A1’	Primary auditory
‘A2’	Secondary auditory
‘CCA’	Anterior cingulate cortex
‘CCP’	Posterior cingulate cortex
‘CCR’	Retrosplenial cingulate cortex
‘CCS’	Subgenual cingulate cortex
‘FEF’	Frontal eye field
‘IA’	Anterior insula
‘IP’	Posterior insula
‘M1’	Primary motor cortex
‘PCI’	Inferior parietal cortex
‘PCIP’	Intraparietal sulcus cortex
‘PCM’	Medial parietal cortex
‘PCS’	Superior parietal cortex
‘PFCCL’	Centrolateral prefrontal cortex
‘PFCDL’	Dorsolateral prefrontal cortex
‘PFCDM’	Dorsomedial prefrontal cortex
‘PFCM’	Medial prefrontal cortex
‘PFCORB’	Orbital prefrontal cortex
‘PFCPOL’	Prefrontal polar cortex
‘PFCVL’	Ventrolateral prefrontal cortex
‘PHC’	Parahippocampal cortex
‘PMCDL’	Dorsolateral premotor cortex
‘PMCM’	Medial (supplementary) premotor cortex
‘PMCVL’	Ventrolateral premotor cortex
‘S1’	Primary somatosensory cortex
‘S2’	Secondary somatosensory cortex
‘TCC’	Central temporal cortex
‘TCI’	Inferior temporal cortex
‘TCPOL’	Polar temporal cortex
‘TCS’	Superior temporal cortex
‘TCV’	Ventral temporal cortex
‘V1’	Primary visual cortex
‘V2’	Secondary visual cortex
‘VACD’	Dorsal anterior visual cortex
‘VACV’	Ventral anterior visual cortex
‘Pulvinar’	Pulvinar thalamic nucleus
‘ThalAM’	Anteromedial thalamic nucleus

**Table 2:** Cross correlations of seed regions.

Correlations Computed from Simulated Data						
	CCP	FEF	PCI	PCIP	PFCM	VACD
CCP	+	−	+	−	+	−
FEF	−	+	−	−*	−	+
PCI	+	−	+	−	+	−
PCIP	−	−*	−	+	−	+
PFCM	+	−	+	−	+	−
VACD	−	+	−	+	−	+

Correlations Obtained from Fox et al. [3]						
	PCC	FEF	LP	IPS	MPF	MT+
PCC	+	−	+	−	+	−
FEF	−	+	−	+	−	+
LP	+	−	+	−	+	−
IPS	−	+	−	+	−	+
MPF	+	−	+	−	+	−
MT+	−	+	−	+	−	+

Positive correlations are denoted by ‘+’ and negative correlations by ‘−’. ‘^*^’ indicates deviation from experimental findings.

**Figure 2:**
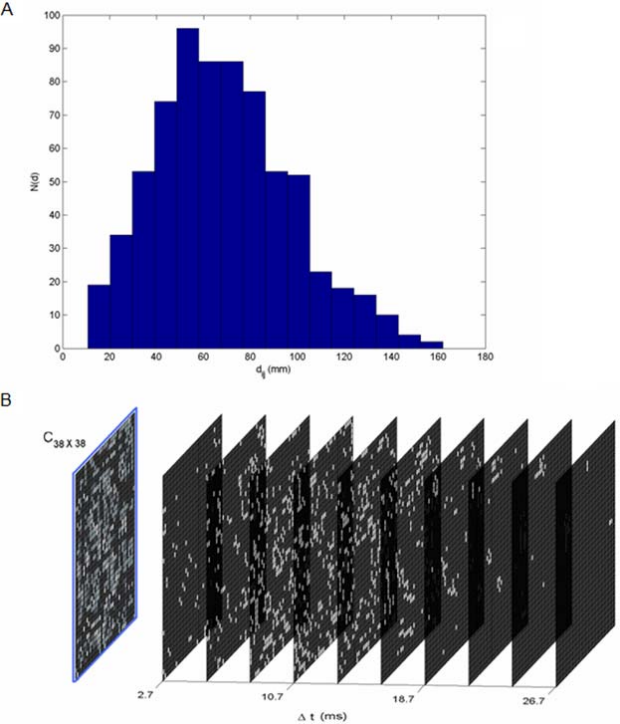
Space time structure of couplings. (A) Distribution of inter-area distances. The time-delays follow identical distribution as we have defined 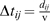, where v is the propagation velocity. (B) Space-time distribution of time-delays. The blue frame shows the spatial connectivity matrix. The nodes having time delay Δt±1.3 ms are snapped to planes denoting time delay Δt for visual clarification. Here we set propagation velocity v = 6 m/s.

To evaluate the temporal aspect of the couplings, i.e., the time delays, we determine these as a function of the spatial position of a given brain area. More specifically, the time delay Δ*t* between any two coupled network nodes is estimated as the ratio d/v, where d is Euclidean distance between two nodes in the three-dimensional physical space and v the propagation velocity along the connecting fibres. The node locations in physical space are chosen to mimic the human brain's geometry and distances based on a standard human atlas. As a consequence, the estimated time delay structure represents a lower estimate. Realistic fibre tracking would generally result in longer pathways than the here estimated shortest distance. Figure 2A illustrates the distribution of the Euclidean distances, which scale linearly with the time delay. The space–time structure of the couplings is illustrated in Figure 2B, in which the individual weights of the connectivity matrix are plotted as a function of the indices of brain areas and their time delay. The projection of all the entries onto the slice with time delay equal to zero yields the anatomical connectivity matrix.

**Figure 3:**
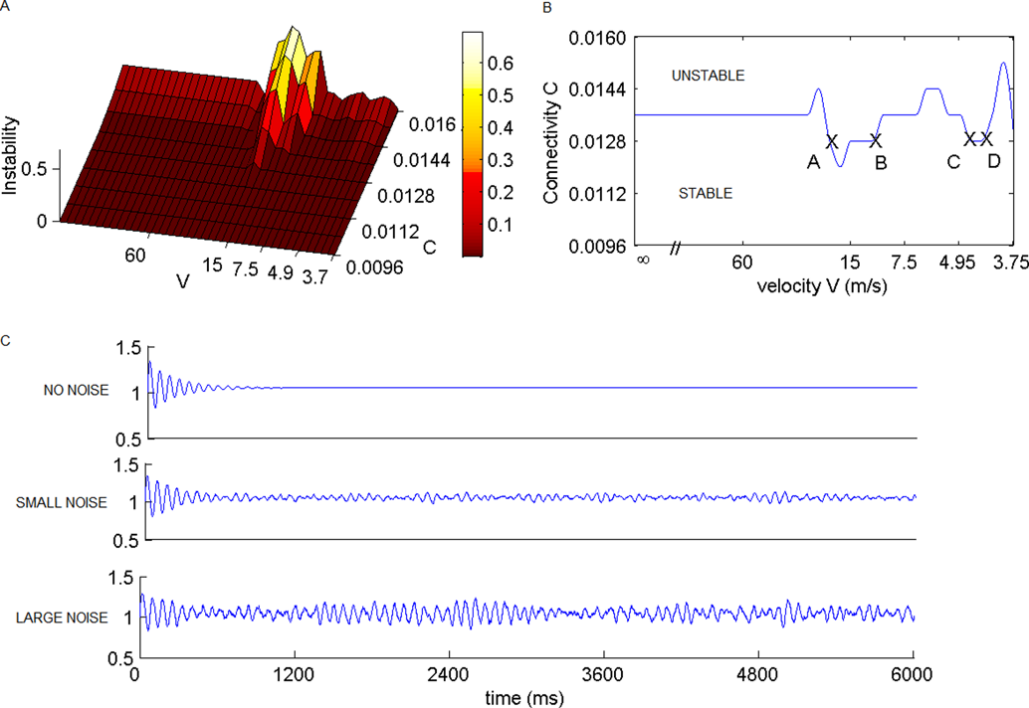
Stability regimes. (A) The degree of instability, equivalent to Re[λ], is plotted as a function of connection strength c and propagation velocity v. (B) The critical boundary, equivalent to Re[λ] = 0, is plotted as a contour line separating unstable and stable regions. (C) Representative time series illustrate the effect of noise upon a single neural population model close to the onset of instability with no noise, small and large noise strength (top-down).

To explore the network dynamics supported by the given space-time structure, we perform simulations for finite signal transmission speeds and investigate the stability of its rest state. We place neuronal oscillators at each network node and couple these via time-delayed interaction terms (see Methods). We have tested multiple oscillator types which are commonly used in neural population modeling including Hopf oscillators [24], Wilson-Cowan systems [25], FitzHugh-Nagumo systems [26],[27], and finally mixed populations of coupled FitzHugh-Nagumo neurons [28], all of which provided similar results. Each population is characterized by a degree of excitability, in which the increase of excitation parameterizes the onset of oscillations emerging from a quiescent state. When the populations are embedded in a network, the network's dynamic repertoire will be shaped by the space-time structure of the couplings. To quantify the total connectivity strength, we introduce a parameter, c, which scales all connection strengths without altering the connection topology of the weight distribution of the matrix, nor affecting the associated time delays Δ*t* = *d*/*ν*. Using this computational framework, we carry out the network simulations with initially identical neuronal population models at each node. The purpose of our simulations is the identification of the critical boundary, which separates the stable and unstable regions of the quiescent state in the parameter space of c and Δ*t*. In its immediate proximity (but still in the stable region), the effect of noise driving the network transiently out of its equilibrium state will be most prominent (see Figure 3C for an illustration of the noise effect upon a single oscillator), and hence easiest to identify. Our results are plotted in Figure 3A, in which the degree of instability of the equilibrium state is plotted as a function of the connection strength, c, and propagation velocity, v. The degree of instability is quantified by the real part of the eigenvalue, Re[λ], from a linear stability analysis of the network's equilibrium state (see Methods). For small values of c, Re[λ]<0 denoting the parameter regions of stability of the equilibrium state, whereas for large values of c the network's equilibrium state is unstable, Re[λ]>0, and displays oscillatory behavior. The two regions are separated by a critical boundary showing a characteristic shape (Figure 3A), of which one segment is more prominent and coincides with the physiologically realistic range of propagation velocities around 5–20 m/s for the adult primate brain (see points A and B in the cross section displayed in Figure 3B). Other points of biological interest (from a clinical and developmental perspective) in the parameter space are the regions indicated by C and D in Figure 3B, which correspond to the transmission speeds of unmyelinated axons, around 1–5 m/s. The emergence of coherent spontaneous fluctuations will be most likely observed in the neighborhood of the critical boundary, since farther away from the boundary all oscillations are either strongly damped or display high amplitude oscillatory behavior, which resembles pathological (e.g., epileptic) activity. Before we proceed to an analysis of the network dynamics, we test the sensitivity of the critical boundary to manipulations of the network architecture in order to gain confidence in its validity (see supplementary materials). To account for errors in the anatomical connectivity, we introduce a distribution of the connection weights, but preserve the general connection topology of the matrix. Further, the impact of parameter heterogeneity of the neuronal populations is assessed by introducing a distribution in their excitability. In all cases, the characteristic shape of the critical boundary (see Figure 3B) proves robust against surprisingly large variations (Figure S7 – weight perturbations; Figure S8 – excitability parameter perturbations). However, when the network is rewired, i.e., changing the connectivity without preserving its connection topology, then the critical boundary disintegrates rapidly (Figure S9). These findings show that the critical boundary displayed in Figure 3A and 3B may be generic for the connection topology of the primate connectivity matrix.

**Figure 4:**
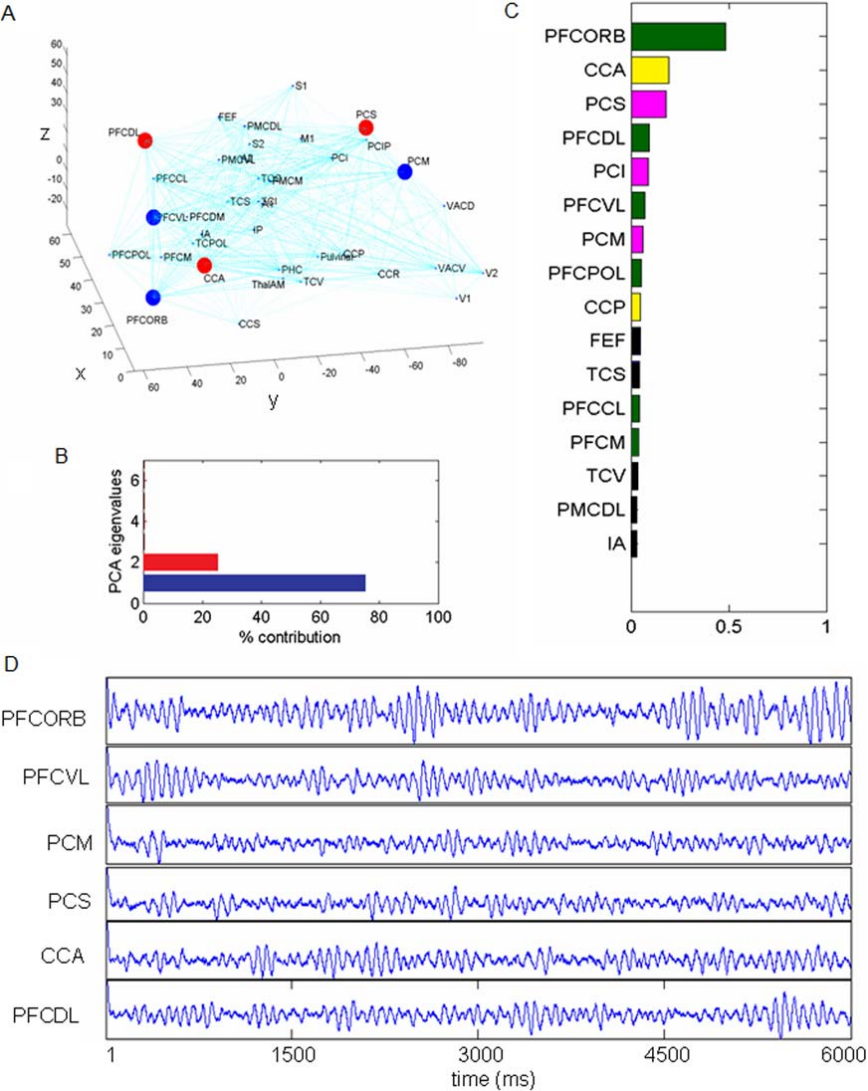
Results of PCA of the network dynamics close to the instability (point B in Figure 3B). (A) The first three dominant areas in the subnetworks as identified by the PCA modes, 1st mode (blue) and 2nd mode (red). The nodes are plotted in the physical space (units in mm) with locations extrapolated for the human. PFCORB, orbital prefrontal cortex; PFCVL, ventrolateral prefrontal cortex; PCM, medial parietal cortex; PCS, superior parietal cortex; CCA, anterior cingulated cortex; PFCDL, dorsolateral prefrontal cortex. (B) The percentile contribution of the first six principal components. The total variance of the first two components is 99.995%. (C) The power of the leading spatial contributions of the first two subnetworks (quantified by PCA) is plotted (normalized per subnetwork). Individual areas are highlighted using the same color coding as in Figure 1C. (D) Time series shown for the rest state subnetwork nodes from a simulation with noise.

To perform a spatiotemporal analysis of the network dynamics, we identify the dominating sub-networks involved in the ongoing transient oscillatory dynamics. We implement the network parameter settings according to point B close to the instability in Figure 3A. Results for other representative parameter settings are presented in the supplementary materials. Our challenge here is to extract the network nodes contributing the most variance to the network dynamics, because these nodes will be the most visible in experimental data. Mathematically speaking, we wish to identify a linear vector space spanned by *n* vectors *ψ_k_*, where *n* is the dimension and typically smaller than the total dimension of the network (in the present network the total dimension is 38). These vectors span the directions of a subspace, in which the network is most sensitive to perturbations and noise. Equivalently, these vectors can be considered to be network patterns or network modes of operation. In this subspace most of the variance of the network dynamics is contained and define the dynamic repertoire of the sorts of the behaviors the network is capable to perform following a perturbation. In other words, the activity of the *i*th network node *u*(*i*,*t*) can be written as 
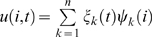
, where *t* is the time and *ξ_k_*(*t*) is the time dependent coefficient capturing the dynamics of the *k*th network pattern *ψ_k_*. The contribution of the *i*th network node is given by *ψ_k_*(*i*). To identify and quantify the contributions of the subspace, we perform the following procedure (see Methods for details): When the network dynamics relaxes into its equilibrium state, we perform a small parameter change towards the unstable region. A typical time series plot is shown in Figure S1. As a consequence of this minimal parameter change, the previously least stable network modes cross the critical boundary first, become unstable and grow with the fastest growth rate. The mathematical basis thereof is the center manifold theorem [29]. As a consequence, only the unstable network modes are present during the transition. Of course, after the transition the nonlinearities and all the network modes become relevant for the network dynamics. During the transition, though, we use a sliding temporal window analysis and perform a Principal Component Analysis (PCA) to identify the dominant network modes (see Figure 4). We find that only two network modes *ψ_k_* contribute to the transient dynamics.

**Figure 5:**
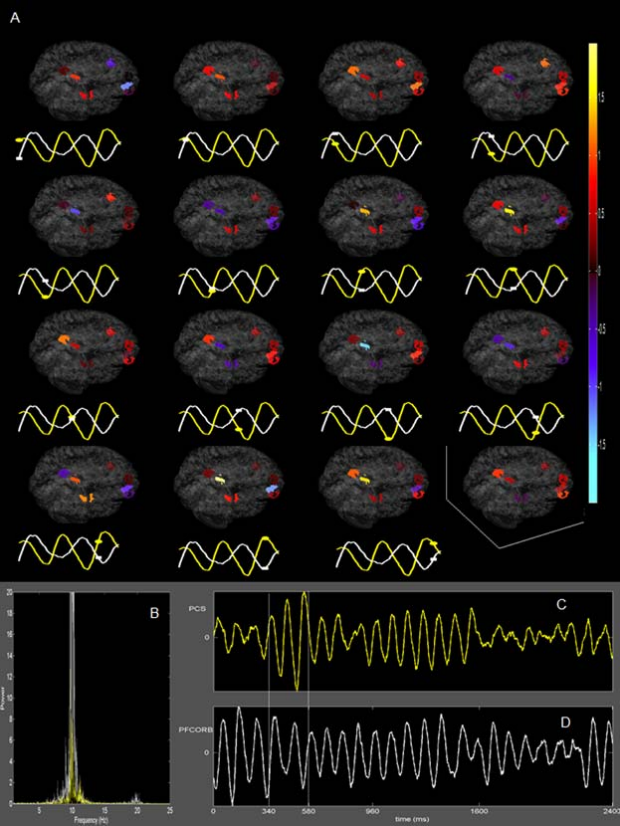
Identification of relevant nodes in resting state network. (A) Temporal evolution of resting state network nodes. Time series at bottom depicts the window of interest for areas PCS (white) and PFCORB (yellow) and markers correspond to instance at which activity is shown on the cortical surface. (B) Fourier power spectra of PCS (white) and PFCORB (yellow) show dominant peak at ∼10 Hz. (C) Time series of neural activity at PCS. (D) Time series of neural activity at PFCORB. Time evolution in (A) is shown for the window marked by vertical lines, 340–580 ms.

The nodes of both networks *ψ_k_*(*i*) are ordered according to power (see Figure 4C). We find that prefrontal, parietal and cingulate cortices rank highest in this ordering scheme and hence contribute most to the two network patterns present during the transient of the instability. We confirm our findings by performing a complete computational network simulation with noise just below the critical boundary and verify that these sub-networks are most commonly present during the transient oscillations of rest state activity. Exemplary time series for the network nodes in the presence of noise are shown in Figure 4D and resemble the characteristic transient and spindle-like time courses with dominant frequencies of 8–12 Hz known from real human resting EEG [30]. To illustrate the spatiotemporal nature of the network dynamics during such an intermittent spindle, we plot a sequence of activation patterns in a cortical surface-based coordinate system for 240 ms in Figure 5.

**Figure 6:**
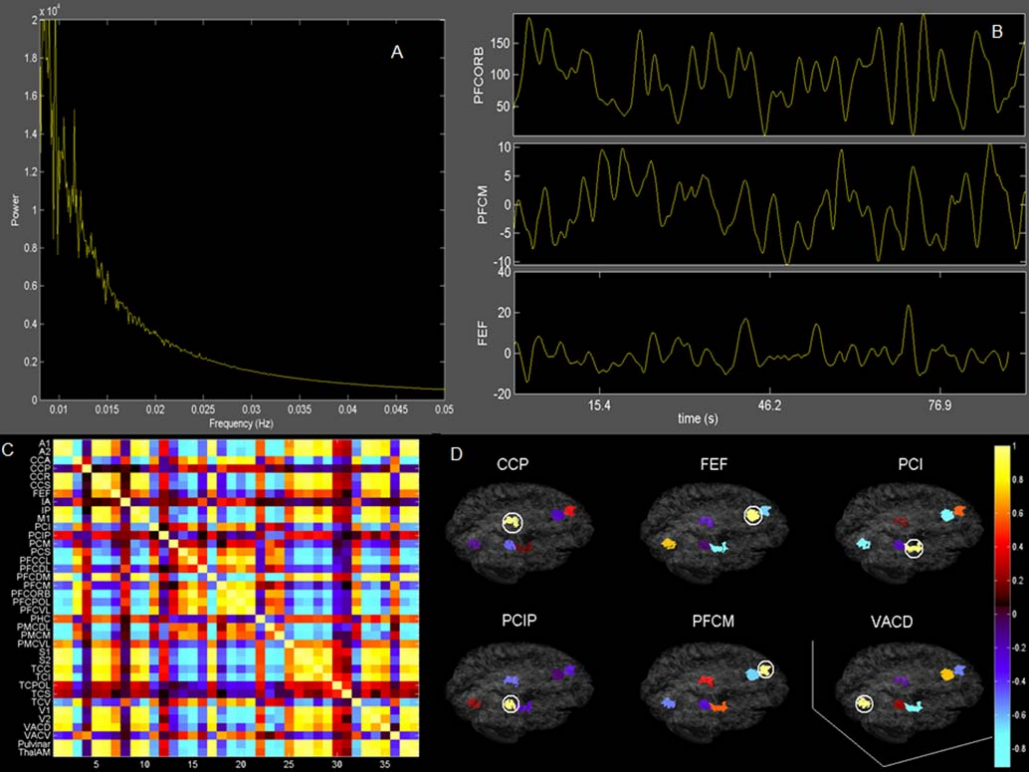
Analysis of BOLD signal activity. (A) Fourier power spectrum of the BOLD signal corresponding to PFCORB node. (B) BOLD signal time series shown for PFCORB, PFCM, FEF. (C) 38×38 correlation matrix computed from the simulated BOLD signals. (D) BOLD signal activity for 6 regions corresponding to Fox et al. is shown.

To test for the emergence of ultra-slow oscillations in the hemodynamic response, we implement the Balloon-Windkessel model [31] and compute the blood oxygen level dependent (BOLD) signal for each of the 38 network nodes (see Methods). A representative time series for the same parameter settings as in Figure 3 (corresponding to point B in the parameter space) is shown in Figure 6. The BOLD time series and their power spectrum show clearly the presence of frequency components in the ultra-slow range of 0.1 Hz. A systematic increase of the transmission speed *v* and hence a reduction of the time delays in the space-time structure results in a reduction of the power in the ultra-slow frequency band. Since our PCA analysis of the neural network dynamics showed the presence of two dominating network patterns,*ψ_k_*, we expect correlated and anti-correlated patterns of activity (captured by the sign of *ψ_k_*(*i*)) on multiple scales, including the one of the BOLD signals. To test for the emergence of anti-correlated networks as reported in Fox et al. [3], we compute the 38×38 cross correlation matrix of the BOLD signals (see Figure 6) and find that mostly positive correlations are present amongst the dominant network nodes as identified in Figure 4., together with various anti-correlated nodes and networks comprising other regions. To perform a more detailed and semi-quantitative comparison with the Fox et al. study [3], we reproduce their analysis. Fox and colleagues chose six predefined seed regions and computed the correlations against all other regions. The seed regions included three regions, referred to as task-positive regions, routinely exhibiting activity increases during task performance, and three regions, referred to as task-negative regions, routinely exhibiting activity decreases during task performance [3]. Task-positive regions were centered in the intraparietal sulcus (IPS; in our notation: PCIP (intraparietal sulcus cortex)), the frontal eye field (FEF) region (same in our notation), and the middle temporal region (MT; in our notation this area is part of VACD (dorsal anterior visual cortex)). Task-negative regions were centered in the medial prefrontal cortex (MPF; in our notation this area corresponds mostly to PFCM (medial prefrontal cortex) and to a lesser extent to PFCPOL (prefrontal polar cortex)), posterior cingulate precuneus (PCC; in our notation CCP (posterior cingulate cortex), but note that the precuneus comprises also our medial parietal cortex PCM), and lateral parietal cortex (LP; in our notation PCI (inferior parietal cortex)). We compute the cross correlations of the seed regions from our simulated data set and illustrate our findings in a surface-based coordinate system in Figure 6. For ease of comparison with the experimental findings in [3] we identify in Table 2 the sign of the cross correlations in experimental and simulated data. Since the cross correlation matrix is symmetric and the diagonal always positive, there remain 15 relevant cross correlations. Notably we find that all cross correlations except one (PCIP-FEF) have the same sign and hence show good correspondence between experimental and simulated data. To underscore further the importance of the transmission delays for biological realism, we perform the identical correlation analysis for a network with infinite transmission speeds (see Figure S10) and find that the cross correlations break down as the transmission speed increases (see Table S1). In particular, out of 15 possible cross correlations, only 7 are captured correctly.

## Discussion

Various mechanisms for the genesis of rest state activity have been put forward including pacemaker oscillators [12],[13], filters [14] and emergent deterministic network dynamics [15]–[20]. The mechanism proposed in this paper lies in between the latter two: noise, omnipresent in any biological system, aids in the sampling of the flow in the vicinity of the brain network's stable equilibrium state. This sampling is reflected in the well-known waxing and waning of EEG and MEG waves during rest. In our computational model, the flow around the equilibrium state is captured by the emergent large scale network dynamics; more specifically, we have demonstrated that the space-time structure of the network's connectivity shapes the flow and actually gives rise to the emergence of coherent fluctuations on a wide range of scales from the ultra slow range <0.1 Hz to high frequencies <100 Hz.

To strengthen evidence that the temporal aspect of the couplings does shape the spatiotemporal dynamics, we scrambled the original time delays under preservation of the actual delay values. The actual spatial aspect of the couplings, i.e., the anatomical connectivity, was kept constant. When performing the same sliding window analysis leading to the results in Figure 5, the resulting emergent networks show different spatial configurations (see Figure S2 for a particular example of scrambled temporal couplings). Different scrambling always results in different emergent network configurations. Furthermore, in a network of identical neuronal populations with instantaneous couplings (no time delay), the anatomical connectivity is the only distinguishing factor amongst the nodes and hence determines the network dynamics. This is illustrated in Figure S3, where the emergent network dynamics is dominated by the area PFCORB. For increasing values of propagation velocity, v, the rest state networks engage the parietal and cingulate areas for v = 5–10 m/s (Figure 4 and Figure S4); upon further decrease of velocity to v = 1 m/s (points C,D in stability diagram Figure 3B) corresponding to unmyelinated fiber transmission speeds, the rest state networks disengage the parietal components and a set of prefrontal areas is distinctly active (Figure S5 and Figure S6). In the various scenarios considered here, the prefrontal areas generally show the largest contributions due to their large degree of connectivity. It shows that the temporal aspects of the coupling will never override the anatomical connectivity, however, as the temporal aspects of the couplings vary, the relative contributions of the nodes change. These changes in the spatial configuration of the resting-state patterns as a function of transmission speed suggest relevance for development and potentially have clinical implications in diseases, in which degradation of myelination is involved. Recent research on rest state activity in infants establishes a partial overlap of the rest state networks with the counterparts in adults, however with an absent component along the posterior-anterior direction [32]. In the adult brain resting-state activity shows a functional correlation both across hemispheres and across brain regions that are spatially separated along the anterior–posterior direction [3],[5],[33]. Our findings regarding the reorganization of the space-time structure of the connectivity explain the difference in spatial network configurations. Indirect anatomical support for our hypothesis is also provided by diffusion tensor MR imaging studies, which revealed a significantly lower anisotropy index in the inferior longitudinal fasciculus, inferior fronto-occipital fasciculus, and superior longitudinal fasciculus compared with the detected degree of anisotropy in the interhemispheric callosal fibers [34]. These findings suggest that the white matter tracts supporting functional connectivity in the anterior–posterior direction are less well developed in the infant brain than the tracts supporting transcallosal functional connectivity [35]. It is worth to reemphasize that our results are obtained for a range of conduction velocities that is in the physiological range. The parameters of the neural population model at each node are constrained to a range to reflect a biologically realistic dynamics in response to a single stimulus. This constraint determines the temporal scale, whereas the spatial scale follows from the locations of the network nodes in the three-dimensional physical space (see Methods). As a consequence, the spatiotemporal scales for the resting state dynamics are fixed within a certain range and the freedom for parameter adjustment is limited. Does it mean that the physiologically observed conduction delays have been somehow selected during development to generate appropriate resting state dynamics? At this stage, the answer is not obvious.

Here we showed that the inclusion of time delays into the space-time structure of the connectivity results in the recruitment of parietal and cingulate cortex for biologically realistic transmission speeds. In contrast, Honey et al. [20] introduced an increased degree of complexity into their network model by utilizing a chaotic dynamics for the brain areas. Their connectivity is also based on biologically realistic primate (though limited to visual and sensorimotor) connectivity, but their assumed transmission speeds are infinite resulting in instantaneous communication within the network. In this configuration, the authors identify BOLD network activations which favorably compare to characteristic rest state networks. Hence the question arises, whether we really need to consider time delays on the order of 10–100 ms when studying BOLD signal fluctuations on the order of <0.1 Hz. After all it would be a computationally most desirable simplification if the time delays could be neglected, since network computations involving time delays are numerically costly. However, since the BOLD signal (in our current understanding of the neurovascular coupling) is generated by the local neural dynamics, which itself evolves on multiple scales including the time scale of signal transmission between areas, neglecting the time delay does not seem permissible. In other words, it is not the BOLD signals on the slow time scale that interact with each other across areas (in which case the neglect of time delay would be justified), but the neural signals evolving on faster time scales. Neither does the chaoticity of the network nodes in Honey et al. [20] substitute for the time delays, but rather introduces another component to a network's node dynamics which we did not address. Our findings hold strictly only if the network nodes display damped oscillatory dynamics in absence of connectivity.

In conclusion, we have demonstrated that the space–time structure of the couplings between brain areas plays a critical role in the functional organization of the emergent network dynamics at rest. On this basis and in the presence of noise, the genesis of a variety of rest state dynamic phenomena including multi-scale oscillations, spatial configurations of networks and some effects of developmental changes can be understood.

## Methods

We quantified the anatomical connectivity using graph theoretical measures [23] where the in-degree and out-degree are the number of incoming and outgoing connections to/from a node. The degree is the sum of in- and out-degree. The clustering coefficient is the number of all existing connections between a node's neighbors divided by all such possible connections. The betweeness centrality is the fraction of the shortest path between any two pairs of nodes passing through a particular node.

The network model with the coupling term of strength *c* is implemented as:
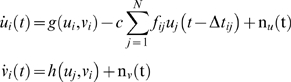
(1)where *u_i_*, *ν_i_* are the state variables of the *i*th neural population and *f_ij_* is the connectivity matrix. White Gaussian noise n*_u_*(t), n*_ν_*(t) is introduced additively. The functions *g* and *h* are based on FitzHugh-Nagumo systems [26],[27] with 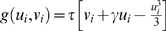 and *h*(*u_i_*,*ν_i_*) = −(1/*τ*)[*u_i_*−*α*+*bν_i_*], and α = 1.05, β = 0.2, γ = 1.0, τ = 1.25. For the stability analysis (no noise) we employed Matlab DDE23 to solve the coupled delay differential equations. The coupled delay differential equations with additive noise were solved in Matlab by a simplified and faster algorithm. More specifically, we employed a standard fourth order Runge-Kutta method for integrating the intrinsic Fitz-Hugh Nagumo dynamics while the coupling and the stochastic terms were integrated using Euler method. The step size for the simulation was 0.001 and we confirmed that no better convergence of solution was achieved using smaller step sizes to ensure numerical convergence.

The time delays 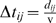 are computed from the Euclidean distance matrix *d_ij_* of the locations of the brain areas *i* and *j*. To do so, the three-dimensional regional map locations were converted to approximate Talairach stereotaxic atlas locations by first identifying the mapping of regional map locations as designated on the human brain to the anatomical locations in Talairach space using the Anatomical Automatic Labeling (AAL) image provided by Tzourio-Mazoyer et al. [36]. Once the approximate location was identified in the AAL brain, the coordinate for the centre of the AAL region was used for the location of the corresponding regional map location. Each region was represented as a surface composed of a sufficient number of triangles. To obtain the triangulation, a T1-weighted MR image from a single human subject was segmented in grey and white matter compartments and the cortical surface represented as a triangular net using the CURRY software package (Compumedics Neuroscan, Ltd). The T1 image was co-registered to a standard MRI atlas (MNI305, [37]) using a 12-parameter affine transform with sinc interpolation as implemented in SPM99 (see http://www.fil.ion.ucl.ac.uk/spm/ and [38]). The transform matrix from the co-registration was then applied to the triangulated cortical surface to the MRI atlas.

The stability diagram for the network in Equation (1) is obtained by linear stability analysis leading to the characteristic equation
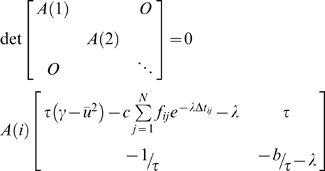
where ū is the fixed point solution. The eigenvalue λ has 2N non-trivial roots with
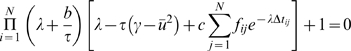
The equilibrium state is stable if all eigenvalues λ have negative real parts, Re(λ)<0, which were found numerically. The stability diagrams in Figure 3 were constructed using this procedure. We also cross-validated the presence of negative real parts of the eigenvalues by direct numerical simulations of Equation 1.

We obtain activity at different areas by simulating Equation 1 for parameter values indicated in the stability diagram. The parameters are chosen to lie on or just below the critical boundary of stable and unstable regions. Network data are simulated for numerical values of parameters in the stable region. Once the network dynamics settles into its equilibrium state (see Figure S1), the coupling parameter c is increased just beyond the critical boundary. We use the smallest increase of c possible given the discretization of the parameter space. As a consequence, now in the unstable regime, the network dynamics increases towards high-amplitude oscillations. A typical time series plot is shown in Figure S1. Using a sliding temporal window of 500 ms width, we perform a Principal Component Analysis (PCA) during the transient as the oscillations increase. The local Center Manifold Theorem guarantees that the network modes with the largest positive real part of the eigenvalue grow fastest and hence dominate the transient initially. Hence, the eigenvectors of PCA span a linear vector space, in which the dominant network modes will be represented. In other words, the networks implicated in rest state activity will be a linear superposition of the PCA eigenvectors. Then the spatiotemporal data can be decomposed as:
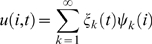
where the *k*th PCA eigenvector *ψ_k_* spans a spatial network. During all transients observed in our simulations, the first two PCA eigenvectors contribute together at least 99.995 percent (see Figure 4B). Hence it does suffice to represent the entire transient dynamics by the first two PCA eigenvectors. Since, in a given PCA eigenvector *ψ_k_*, each node is multiplied by the same time-dependent coefficient *ξ_k_*(*t*), the magnitude of the *i*th vector element will scale the resulting contribution of the *i*th node to the network dynamics. The most dominant nodes of these two networks *ψ_k_*(*i*) are then identified through an ordering process: we compute *ψ_k_*(*i*)^2^ for all nodes *i* and both network modes *k* = 1, 2 and order these according to power (see for instance Figure 4C). There is no hard criterion to identify a threshold for the inclusion of nodes in a network. For reasons of clarity, we choose to show the first three dominating nodes for each eigenvector in Figure 4A, which corresponds to at least 90% of the power per eigenvector in all cases.

To relate the simulated neural activity to recent fMRI studies, we have generated BOLD signal for each regions by using a hemodynamic model. This model combines the Balloon/Windkessel model comprised in venous volume and deoxyhemoglobin content with a linear dynamical model of how synaptic activity causes changes in regional cerebral blood flow [31]. For each region, neural activity causes an increase in a vasodilatory signal inducing blood flow, which changes blood volume and deoxyhemoglobin content. The BOLD signal is given by a volume-weighted sum of extra- and intra vascular signals as the function of volume and deoxyhemoglobin content. The local neural activity, which is taken to be the absolute value of the time derivative of the output occurring by our network model in each brain region, is used as the main model input to estimate a BOLD signal. For the analyses, the global mean signal (average over all regions) has been regressed out from the single BOLD time series. All parameters regarding blood flow, deoxyhemoglobin content, and vessel volume in the model equation are taken from [31].

## Supporting Information

Figure S1 A representative time series plotted for all nodes, shown for the fast variable u, as the system undergoes a stable-unstable transition. The initial parameters correspond to stable region and at a time indicated by arrow, the propagation velocity has been switched to make the system unstable.
(0.09 MB TIF)

Figure S2 Results of PCA as instability sets in at the edge marked B in Figure 2A for scrambled delays. (a) Subnetwork as identified by dominant PCA modes, 1st mode (blue) and 2nd mode (red) with a combined total variance of 99.989%. (b) The percentile contribution of the principal components. (c) The normalized power of the first two dominant spatial modes is shown for the largest components.
(0.09 MB TIF)

Figure S3 PCA of spatiotemporal data in absence of time delay (v→∞). (a) Subnetwork as identified by dominant PCA modes, 1st mode with a variance of 99.92%. (b) The percentile contribution of the principal components. (c) The normalized power of the dominant spatial mode is shown for the largest components.
(0.10 MB TIF)

Figure S4 Results of PCA as instability sets in at the edge marked A in Figure 2A. (a) Subnetworks as identified by dominant PCA modes, 1st mode (blue) and 2nd mode (red) with a combined total variance of 99.996%. (b) The percentile contribution of the principal components. (c) The normalized power of the first two dominant spatial modes is shown for the largest components.
(0.10 MB TIF)

Figure S5 Results of PCA as instability sets in at the edge marked C in Figure 2A. (a) Subnetwork as identified by dominant PCA modes, 1st mode (blue) and 2nd mode (red) with a combined total variance of 99.858%. (b) The percentile contribution of the principal components. (c) The normalized power of the first two dominant spatial modes is shown for the largest components.
(0.10 MB TIF)

Figure S6 Results of PCA as instability sets in at the edge marked D in Figure 2A. (a) Subnetwork as identified by dominant PCA modes, 1st mode (blue) and 2nd mode (red) with a combined total variance of 99.989%. (b) The percentile contribution of the principal components. (c) The normalized power of the first two dominant spatial modes is shown for the largest components.
(0.10 MB TIF)

Figure S7 The results of the stability analysis are robust against weight perturbations in the connectivity matrix. A cross section of the stability diagram in Figure 2A is shown for c = 0.016. The weights wij of the connection matrix are perturbed randomly, such that the actual weight, wij ±E, varies with square error, E (color-coded in legends). With increasing perturbation strength, the degree of instability grows, but the actual shape of the curve does not change.
(0.08 MB TIF)

Figure S8 The results of the stability analysis are robust against perturbations of the excitability parameter, a. A cross section of the stability diagram in Figure 2A is shown for c = 0.016. The excitabilities are perturbed randomly for each node dynamics, such that the actual excitability, a ±E, varies with square error, E (color-coded in legends). With increasing perturbation strength, the degree of instability reduces, but the actual shape of the curve does not change and for large perturbation system dynamics tends to become unstable.
(0.08 MB TIF)

Figure S9 Disintegration following change of network topology. A cross section of the stability diagram in Figure 2A is shown for c = 0.016. The network is rewired randomly, where p is the probability of rewiring the existing network. The characteristics of the cross section are lost for small rewiring probabilities and are not regained again.
(0.08 MB TIF)

Figure S10 Correlation matrix computed from the simulated BOLD signals for v →∞. Here, in full analogy to Figure 6, we computed the BOLD signals from the network dynamics for the case when the time delays are negligible, i.e., communication speed between areas is infinite. All other parameters are identical as in Figure 6.
(0.27 MB TIF)

Table S1 Cross correlations of seed regions for v →∞. Correlations computed from simulated data. Cross correlation of seed regions for a network dynamics with negligible time delays. In full analogy to Table 2, positive correlations are denoted by ‘+’ and negative correlations by ‘−’. Circle indicates deviation from experimental findings.
(0.03 MB DOC)
