# Supplementary figures and images for: Noise during Rest Enables the Exploration of the Brain's Dynamic Repertoire
Source: PLoS Comput Biol. 2008 Oct 10;4(10):e1000196. doi: 10.1371/journal.pcbi.1000196 (PMC2551736; doi:10.1371/journal.pcbi.1000196)

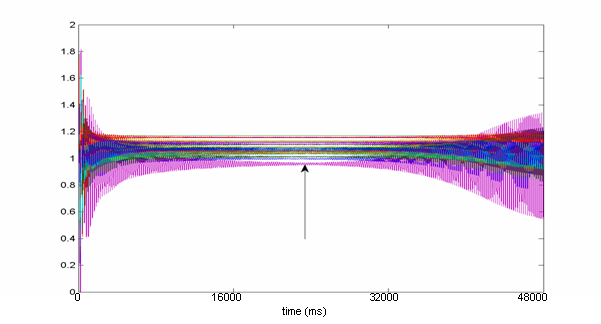

Supplement: Figure S1 — A representative time series plotted for all nodes, shown for the fast variable u, as the system undergoes a stable-unstable transition. The initial parameters correspond to stable region and at a time indicated by arrow, the propagation velocity has been switched to make the system unstable. (0.09 MB TIF) [file pcbi.1000196.s001.tif]

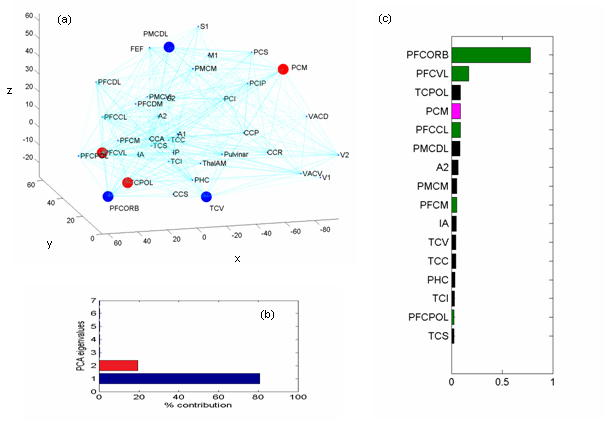

Supplement: Figure S2 — Results of PCA as instability sets in at the edge marked B in Figure 2A for scrambled delays. (a) Subnetwork as identified by dominant PCA modes, 1st mode (blue) and 2nd mode (red) with a combined total variance of 99.989%. (b) The percentile contribution of the principal components. (c) The normalized power of the first two dominant spatial modes is shown for the largest components. (0.09 MB TIF) [file pcbi.1000196.s002.tif]

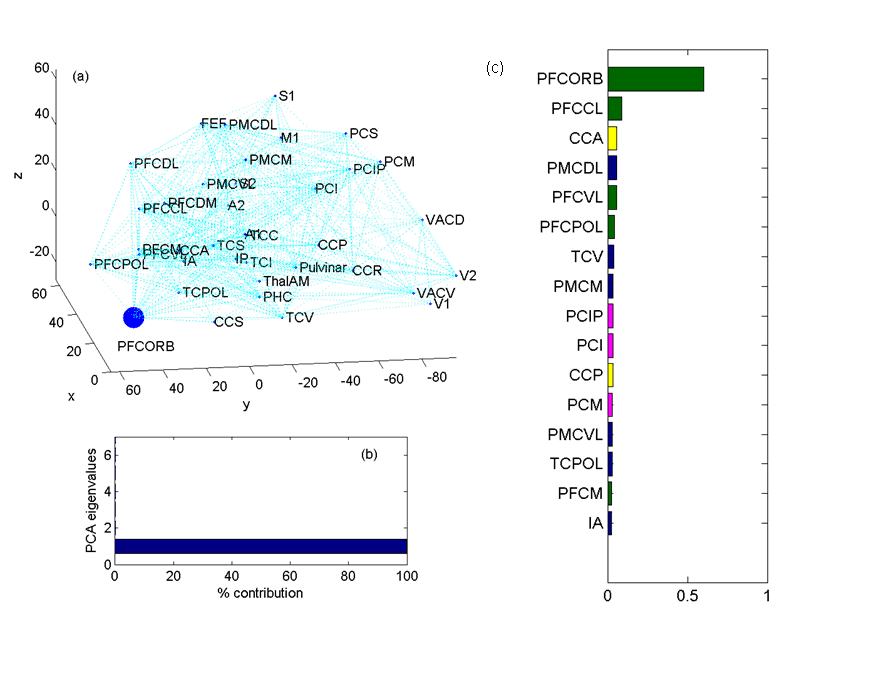

Supplement: Figure S3 — PCA of spatiotemporal data in absence of time delay (v→∞). (a) Subnetwork as identified by dominant PCA modes, 1st mode with a variance of 99.92%. (b) The percentile contribution of the principal components. (c) The normalized power of the dominant spatial mode is shown for the largest components. (0.10 MB TIF) [file pcbi.1000196.s003.tif]

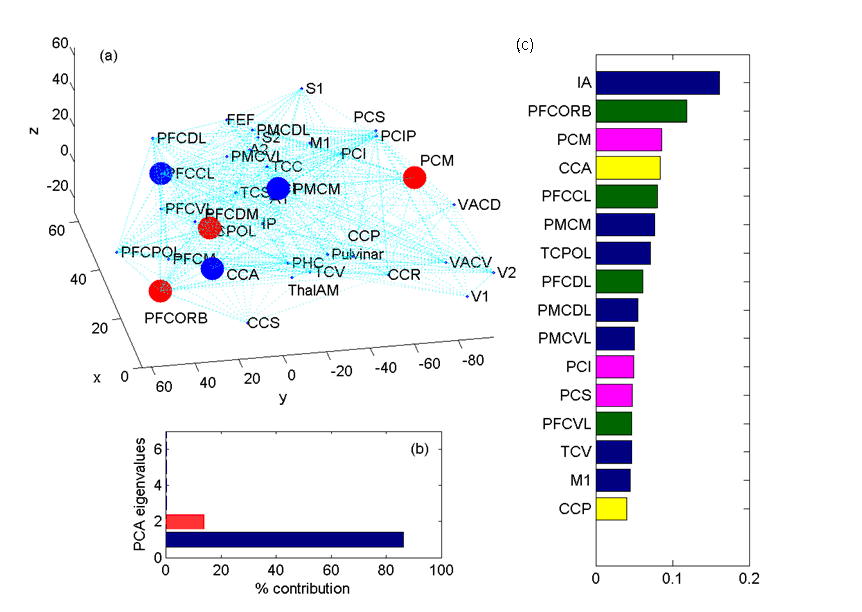

Supplement: Figure S4 — Results of PCA as instability sets in at the edge marked A in Figure 2A. (a) Subnetworks as identified by dominant PCA modes, 1st mode (blue) and 2nd mode (red) with a combined total variance of 99.996%. (b) The percentile contribution of the principal components. (c) The normalized power of the first two dominant spatial modes is shown for the largest components. (0.10 MB TIF) [file pcbi.1000196.s004.tif]

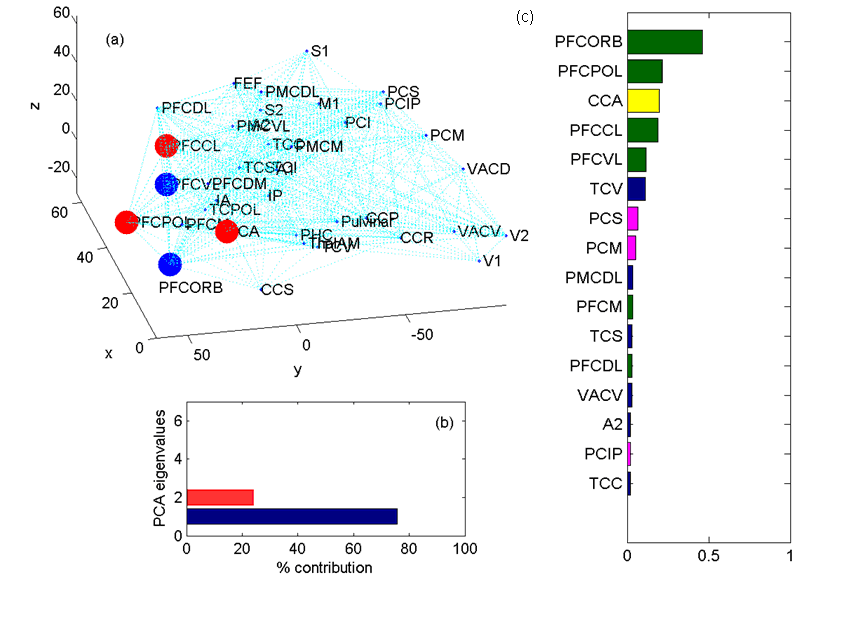

Supplement: Figure S5 — Results of PCA as instability sets in at the edge marked C in Figure 2A. (a) Subnetwork as identified by dominant PCA modes, 1st mode (blue) and 2nd mode (red) with a combined total variance of 99.858%. (b) The percentile contribution of the principal components. (c) The normalized power of the first two dominant spatial modes is shown for the largest components. (0.10 MB TIF) [file pcbi.1000196.s005.tif]

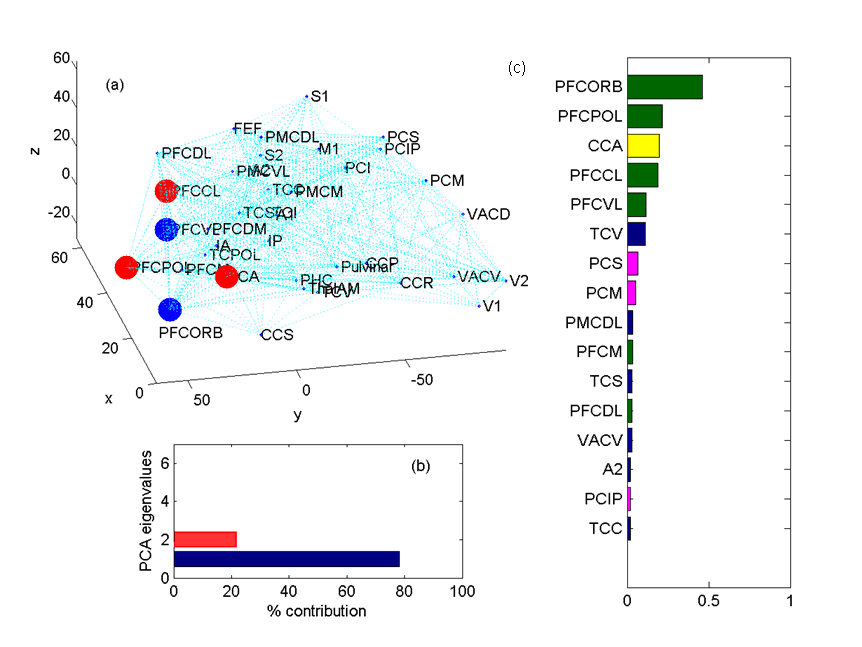

Supplement: Figure S6 — Results of PCA as instability sets in at the edge marked D in Figure 2A. (a) Subnetwork as identified by dominant PCA modes, 1st mode (blue) and 2nd mode (red) with a combined total variance of 99.989%. (b) The percentile contribution of the principal components. (c) The normalized power of the first two dominant spatial modes is shown for the largest components. (0.10 MB TIF) [file pcbi.1000196.s006.tif]

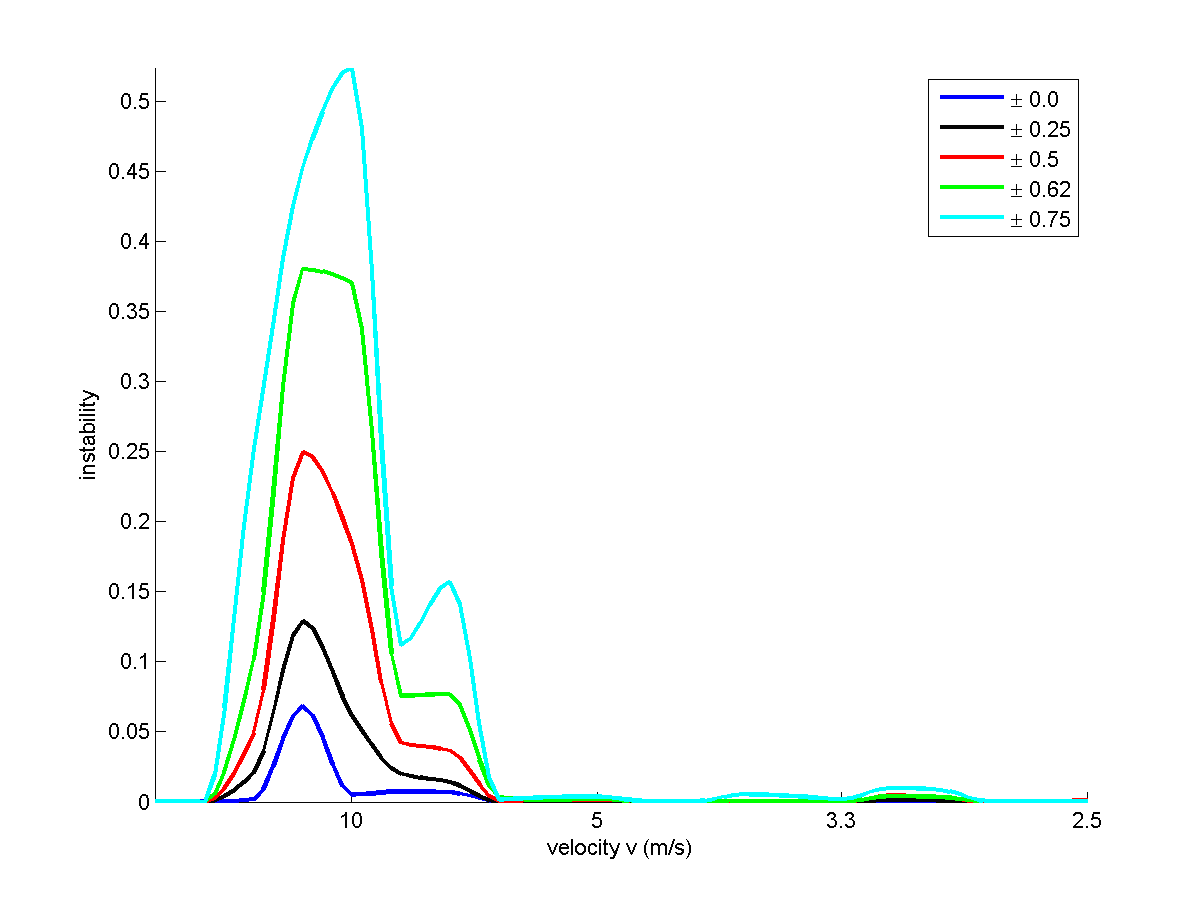

Supplement: Figure S7 — The results of the stability analysis are robust against weight perturbations in the connectivity matrix. A cross section of the stability diagram in Figure 2A is shown for c = 0.016. The weights wij of the connection matrix are perturbed randomly, such that the actual weight, wij ±E, varies with square error, E (color-coded in legends). With increasing perturbation strength, the degree of instability grows, but the actual shape of the curve does not change. (0.08 MB TIF) [file pcbi.1000196.s007.tif]

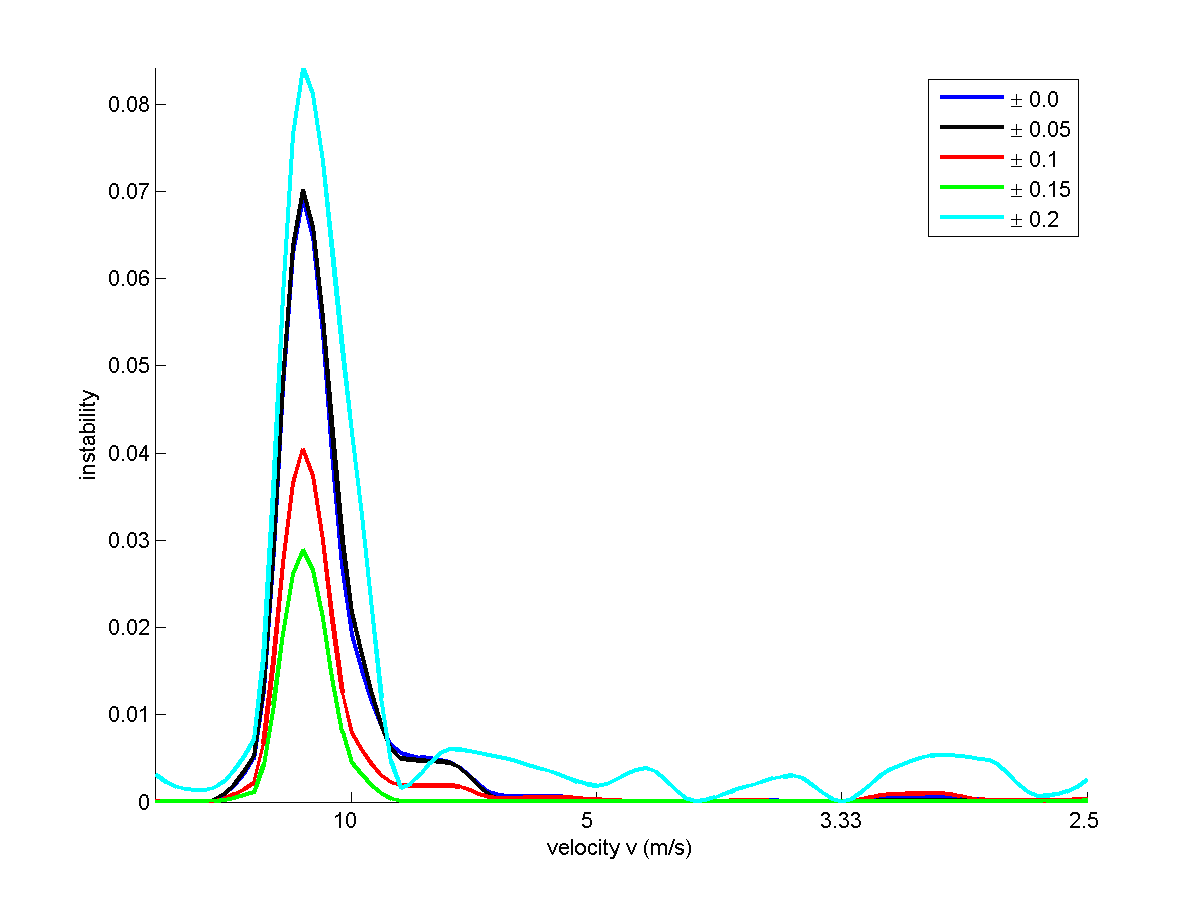

Supplement: Figure S8 — The results of the stability analysis are robust against perturbations of the excitability parameter, a. A cross section of the stability diagram in Figure 2A is shown for c = 0.016. The excitabilities are perturbed randomly for each node dynamics, such that the actual excitability, a ±E, varies with square error, E (color-coded in legends). With increasing perturbation strength, the degree of instability reduces, but the actual shape of the curve does not change and for large perturbation system dynamics tends to become unstable. (0.08 MB TIF) [file pcbi.1000196.s008.tif]

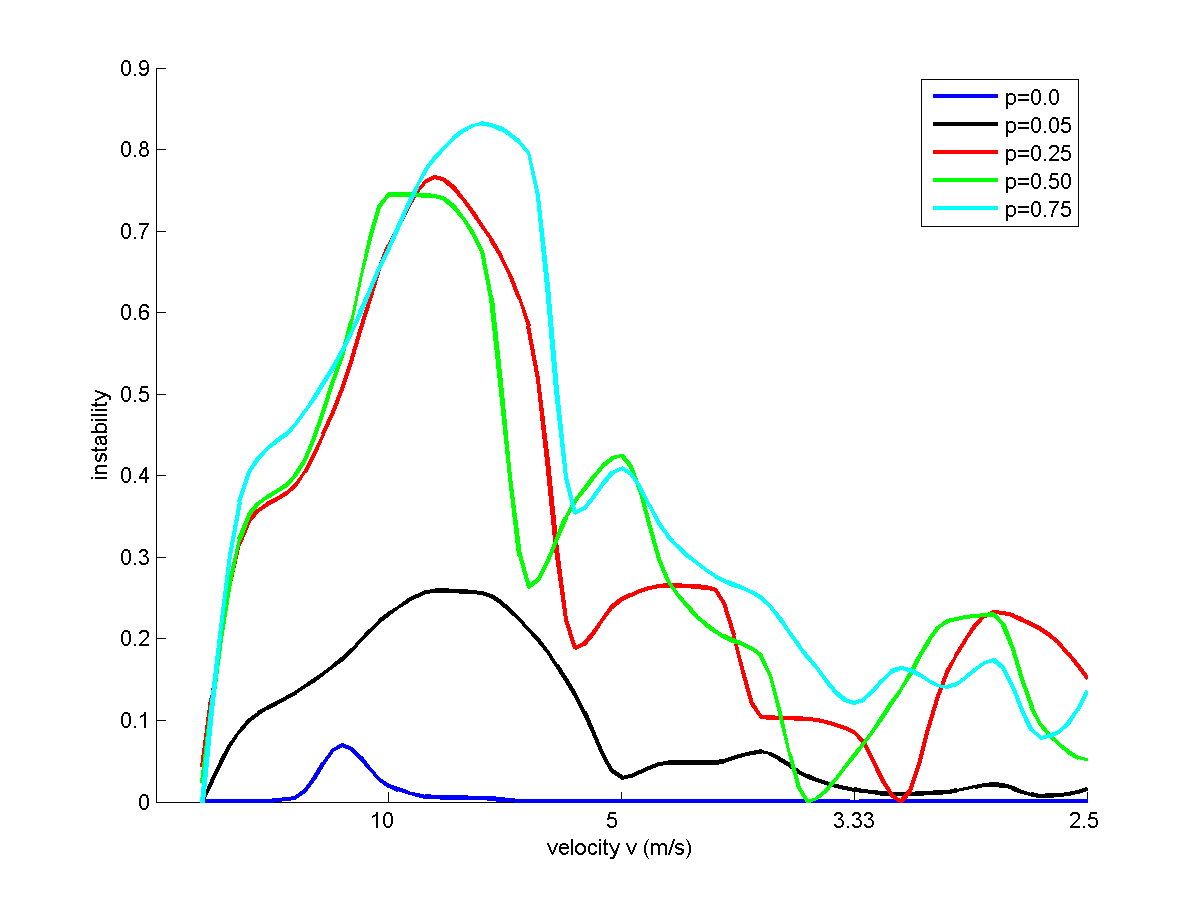

Supplement: Figure S9 — Disintegration following change of network topology. A cross section of the stability diagram in Figure 2A is shown for c = 0.016. The network is rewired randomly, where p is the probability of rewiring the existing network. The characteristics of the cross section are lost for small rewiring probabilities and are not regained again. (0.08 MB TIF) [file pcbi.1000196.s009.tif]

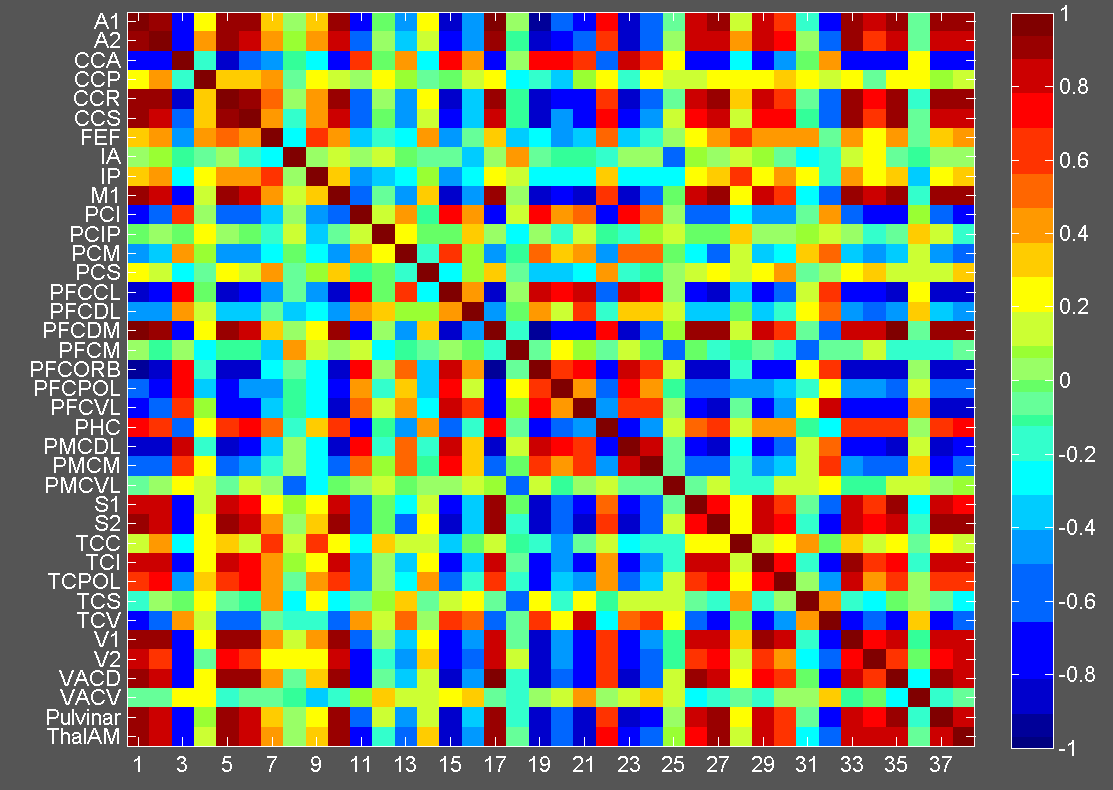

Supplement: Figure S10 — Correlation matrix computed from the simulated BOLD signals for v →∞. Here, in full analogy to Figure 6, we computed the BOLD signals from the network dynamics for the case when the time delays are negligible, i.e., communication speed between areas is infinite. All other parameters are identical as in Figure 6. (0.27 MB TIF) [file pcbi.1000196.s010.tif]
