# Supplementary material for: Noise during Rest Enables the Exploration of the Brain's Dynamic Repertoire
Source: PLoS Comput Biol. 2008 Oct 10;4(10):e1000196. doi: 10.1371/journal.pcbi.1000196 (PMC2551736; doi:10.1371/journal.pcbi.1000196)
Supplement: Table S1 — Cross correlations of seed regions for v →∞. Correlations computed from simulated data. Cross correlation of seed regions for a network dynamics with negligible time delays. In full analogy to Table 2, positive correlations are denoted by ‘+’ and negative correlations by ‘−’. Circle indicates deviation from experimental findings. (0.03 MB DOC) [file pcbi.1000196.s011.doc]

**Table S1: Cross correlations of seed regions for v →∞**

|  | CCP | FEF | PCI | PCIP | PFCM | VACD |
| --- | --- | --- | --- | --- | --- | --- |
| CCP | + | + | - | + | - | + |
| FEF | + | + | - | - | - | + |
| PCI | - | - | + | + | + | - |
| PCIP | + | - | + | + | - | - |
| PFCM | - | - | + | - | + | - |
| VACD | + | + | - | - | - | + |
